# Irrigation with primary wastewater alters wood anatomy and composition in willow *Salix miyabeana* SX67

**DOI:** 10.3389/fpls.2023.1087035

**Published:** 2023-03-02

**Authors:** Ahmed Jerbi, Joan Laur, Kevin Lajoie, Pierre-Paul Gallant, Simon Barnabé, Frederic E. Pitre, Michel Labrecque

**Affiliations:** ^1^ Institut de recherche en biologie végétale, Université de Montréal, Montréal, QC, Canada; ^2^ Montreal Botanical Garden, Research and Development Division, Montréal, QC, Canada; ^3^ Institut d’Innovations sur les Écomatériaux, Écoproduits et Écoénergies à base de biomasse (I2E3), Université du Québec à Trois-Rivières, Trois-Rivières, Canada; ^4^ École de technologie supérieure, Université du Québec, Montréal, QC, Canada

**Keywords:** willow, wastewater, phytofiltration, cell wall composition, hydraulic conductance, sustainable biomass

## Abstract

Traditional treatment of wastewaters is a burden for local governments. Using short rotation coppice willow (SRCW) as vegetal filter has several environmental and economic benefits. Here, we investigated the effect of primary wastewater irrigation on wood structure and composition of the willow cultivar *Salix miyabeana* ‘SX67’ following two years of growth. Compared to unirrigated plants (UI), stem sections of plants irrigated with primary wastewater (WWD) showed an unexpected decrease of hydraulic conductance (K_S_) associated with a decrease in vessel density but not vessel diameter. The majority (86%) of vessels had diameters range groups [20-30[, [30-40[and [40-50[µm and contributed to > 75% of theoretical K_S_, while the group class [50-60[µm (less than 10% of vessels) still accounted for > 20% of total K_S_ regardless irrigation treatments. WWD significantly alters the chemical composition of wood with an increase of glucan content by 9 to 16.4% and a decrease of extractives by 35.3 to 36.4% when compared to UI or to plants irrigated with potable water (PW). The fertigation did also increase the proportion of the tension wood which highly correlated with glucan content. In the context of energetic transition and mitigation of climate change, such results are of high interest since WWD effectively permit the phytofiltration of large amounts of organic contaminated effluents without impairing SRCW physiology.

## Highlights

**-** Primary wastewater irrigation alters willow wood properties;

**-** Plants irrigated with primary wastewater showed a high proportion of tension wood and a decrease of hydraulic conductance (K_S_) associated with lower vessel density;

**-** Fertigation increased glucan content but decreased extractives;

- Glucan content highly correlates with the proportion of tension wood.

## 1 Introduction

Standard treatment strategies of municipal wastewater have a high financial cost. It is also not totally efficient to decontaminate wastewater that can be released untreated or undertreated in the environment with release of high ammonia content within natural water bodies (Jerbi et al., 2020). In Canada, near to 6 trillion liters of municipal wastewater is discharged each year from which an estimated 100-270 billion liters is released into the environment without any treatment, ~1.5 trillion liters receives primary treatment (where suspended solids and some organic matter are removed), ~2.8 trillion liters receives secondary treatment (where organic matter is degraded using biological treatment) and ~1.4 trillion liters receives tertiary treatment (where remaining solids, nutrient and emerging contaminants are removed by a range of polishing steps) (Statistics Canada, 2017).

The use of short-rotation willow coppice (SRWC) as a vegetation filter has potential to both drastically enhance plantation productivity and improve the quality of pretreated wastewater prior to discharge into a water body (Guidi Nissim et al., 2015; Jerbi et al., 2015). Since this approach fulfills by far the willow requirement in term of water and nitrogen, it allows plants to overcome their high evapotranspiration rate and thus improve carbon assimilation and biomass production (Dimitriou and Aronsson, 2004; Dimitriou and Aronsson, 2011; de Miguel et al., 2014; Guidi Nissim et al., 2015). Previous studies have reported the positive effects of wastewater fertigation on various physiological and morphological SRWC parameters: leaf area, leaf N content, chlorophyll content, stomatal conductance, photosynthesis and carbon assimilation, below ground biomass and above ground productivity (Dimitriou and Aronsson, 2011; Guidi Nissim et al., 2015; Jerbi et al., 2015; Lachapelle-T. et al., 2019; Jerbi et al., 2020). Xylem development and the resulting wood characteristics are also strongly affected by environmental parameters, namely water or nitrogen availability (Arend and Fromm, 2007; Hacke et al., 2010; Pitre et al., 2010; Plavcová and Hacke, 2012; Anfodillo et al., 2013; Plavcová et al., 2013, Wang et al., 2019) which are not limited in the present context. Yet very little is known about the effect of municipal effluent fertigation on the structural and chemical biomass composition even though its alterations could not only influence plant physiology (Jerbi et al., 2020) but also biofuel production.

At the anatomical level, wood structure is altered in response to fertigation, the physical structure of vessel and fiber cells (i.e. number per unit area) within the xylem tissue have a great impact on plant function as a whole and on wood hydraulic and mechanical properties (Mellerowicz et al., 2001; Pitre et al., 2007a; Martínez-Cabrera et al., 2011). Specific conductivity (theorical K_S_) and hydraulic conductance are functions of vessel density and stem (Tyree and Zimmermann, 2002). Although larger vessels are likely to improve hydraulic conductance, such conduits result in a lower fraction of supporting tissue and thus lead to a decrease in stem mechanical strength (Poorter et al., 2010). They are also more vulnerable to embolism when exposed to environmental stresses like drought, heat waves and freeze-thaw events (Sperry et al., 2008; Martínez-Cabrera et al., 2011; Plavcová and Hacke, 2012; Hacke et al., 2017), the latter being frequent in remote regions of Canada where the effective deployment of this combined phytotechnology (SRWC + vegetation filter) has the greatest potential.

Wood composition as well as the form of major polymers, lignin, cellulose and hemicellulose, vary also considerably between plant species, genotypes and because of environmental factors (Pitre et al., 2007a; de Souza, 2013). Conducted mostly on poplar, several studies have reported the effects of N fertilization on wood structure (mostly fiber lumen and cell walls) and chemical composition, especially the content of extractives, lignin, glucose, xylose and arabinose (Serapiglia et al., 2009; Ray et al., 2012; Serapiglia et al., 2013b; Wan et al., 2014). Nitrogen availability affects the development of secondary xylem during cell division and differentiation and leads to an alteration of either xylem anatomy and/or wood structural composition (Luo et al., 2005; Pitre et al., 2007a; Pitre et al., 2010) with an increase in cellulose content, a reduced lignin fraction, an increase of the tension wood proportion, a decrease of fiber cell walls and a decline in wood density (Harvey and van den Driessche, 1999; Luo et al., 2005; Pitre et al., 2007a; Pitre et al., 2010; Serapiglia et al., 2013a). Albeit willow cultivation is of equal economic importance, less research has assessed the effect of high N fertilization on the compositional and morphological traits of willow cultivars.

The aim of this study was to investigate the short-term effects of primary municipal wastewater on wood mechanical structure and composition of willow cultivar *Salix miyabeana* ‘SX67’. We test the hypothesis that fertigation with high-N-content wastewater effluent (WWD) would alter the wood composition (i.e. the lignocellulosic structure of fiber cell-wall) as well as the xylem structure with a wider vessel for WWD plants when compared to either unirrigated and/or potable water irrigated plants, thus, enhancing plant water conductance capacity.

## 2 Material and methods

### 2.1 Study site and plant material

The experimental plantation was located in Saint-Roch-de-l’Achigan (45° 50′ 50″N–73° 38′ 27″W), 57 m above sea level, 55 km northeast of Montreal (Quebec), Canada. The regional climate is humid continental with noticeable seasonal temperature variations, warm, humid summers and cold winters. According to the nearest weather station in Assomption (45° 48 34”N–73° 26 05”W), the annual average minimum and maximum temperatures for the period 2003-2017 are respectively 1 ± 12°C and 11 ± 13° C. During the growing period (from May 1 to October 31, 2017), average minimum and maximum temperatures were recorded by in-field meteorological station and corresponded to 9.8 ± 5.6° C and 22.1 ± 6.5° C respectively (Amiot et al., 2020). The average annual precipitation was 1102 mm (2005-2015).

Four hectares of *Salix miyabeana* ‘SX67’ were established in 2008 at a density of 16,000 trees ha^−1^ with 1.8 m and 0.35 m spacing respectively between willow rows and between cuttings within each row (Guidi Nissim et al., 2015; Jerbi et al., 2015). A first experiment aimed at treating secondary municipal wastewater effluent was conducted on the plantation between 2009 and 2012 (Guidi Nissim et al., 2015; Jerbi et al., 2015). The plantation was last coppiced prior the present experiment when plants roots were seven-year-old in the autumn of 2015. In 2016, a randomized block design was set up (plots spatially distinct from those of 2009-2012 study) comprising four irrigation treatments replicated three times: a control without irrigation (UI), irrigation with potable water (PW), and irrigation with two different doses of the same primary effluent wastewater (Lachapelle-T. et al., 2019). Only UI, PW and the lowest dose of wastewater (noted here as WWD) are used in the present study. Nine experimental square plots (3 treatments x 3 plots) of 100 m^2^ were delimited, each containing six rows of willow with the four central rows irrigated. Four randomly chosen plants in each of those latter four rows were identified and served for most of sampling and analysis reported here.

Primary wastewater was obtained from the local municipal wastewater treatment facility and was allowed to rest for at least 24 h in a conventional septic tank prior to irrigation of the plantation with no further chemical or biological treatment. The plantation was irrigated 111 and 163 days respectively for 2016 and 2017 with a daily dose of respectively 14 mm and 13 mm for PW, 10 mm and 12 mm for WWD. The average annual loads of elements given to the WWD plants through fertigation were 594 kg N ha^-1^, 58 kg P ha^-1^, 174 kg K ha^-1^, 1776 kg Ca ha^-1^, 515 kg Mg ha^-1^, 3566 kg Na ha^-1^ and 5491 kg Cl ha^-1^. Experimental setup schematic, characterization of primary wastewater, annual precipitation, loads of water and wastewater as well as the loads of all the nutrients through wastewater irrigation (per year and cumulative) are presented in the supplementary material (**Figure S1**, **Tables S1–S3**), further details on the experiment are described in Lachapelle-T. et al. (2019); Amiot et al. (2020) and Jerbi et al. (2020).

### 2.2 Plant sampling and biomass processing

At the end of the 2017 growing season, all above-ground biomass from the four labelled trees within each plot were harvested, for a total of 36 plants (3 treatments x 12 replicates). All trees were fresh-weighed on the field and random subsamples of stems were oven dried at 105° C for 72 h to assess moisture content. The biomass yields were estimated based on dry matter yields at planting density of 16,000 trees ha^−1^.

### 2.3 Wood density (wood specific gravity)

In August 2017, two 20-25 cm stem sections were collected from the 36 labelled trees. They were immediately fixed in a formaldehyde-acetic acid-alcohol solution (FAA solution: 3.7% formaldehyde, 5% acetic acid and 47% alcohol). Subsections (3-4 cm; one per tree) were labelled, vacuum infiltrated with water for 48 h and used to determine stem volume by water displacement. Wood specific gravity was later calculated from green volume and oven-dry weight at 105° C (Berthod et al., 2015; Brereton et al., 2015).

### 2.4 Microscopy and image analysis

As fertigated trees were more developed than UI and PW trees, stem sections were collected at different stem height (e.g. sections at breast height for UI and PW, but at a higher level for WWD) in order to compare developmentally similar wood (Pitre et al., 2007a). Stems were sectioned where wood had already transitioned to secondary growth before irrigation began, thus allowing analysis of the wood (secondary xylem) that was formed before and during the irrigation period to assess the effect of nitrogen fertilization on growth, development and on histologic wood properties (Pitre et al., 2007a). The diameter of the sections were between 9 and 10 mm.

#### 2.4.1 Stem sectioning and staining

A transverse section of 25 μm thickness of a FAA-fixed stem was made using a rotary microtome (Leica RM2235, Germany). To monitor changes in the relative proportion of lignin and cellulose, the 25 μm cross section was double-stained with 1% aqueous Safranin O to stain lignified cell walls and with 1% Chlorazol Black E in methoxyethanol to staining cellulose G-fiber. Sections were permanently mounted on glass slides with DPX medium (Brereton et al., 2011; Brereton et al., 2012).

#### 2.4.2 Image acquisition and analysis

All 36 slides were scanned digitally using a linear whole slide scanner (Aperio ScanScope CS2, Leica, Germany) at 40x objective magnification. Raw image data were stored in Aperio SVS file format, a multi-layered compressed JPEG (further information on the image format can be found in Shawki et al. (2020)). The slide images varied in size from 0.2 to 0.4 Gb and were first viewed using Leica Aperio Imagescope digital slide viewer version 12 (Leica Biosystems, Aperio) to examine each entire slide for any potential problems that could interfere with analysis, e.g. air/dust spots, partial staining, missing xylem parts. The SVS image slides were then analyzed by a script developed in-house in Fiji image-processing software (www.fiji.sc, ImageJ, (Schindelin et al., 2012)). More details on the script and the image analysis methods are presented in the supplementary material (**Supplementary comment 1**). The Fiji script was run on a super-computing platform so that the SVS file could be opened as a big tiff file (approximately 20 Gigabytes each) and the whole image analyzed in smaller fragments to examine the various xylem features (which are described in the section below). The technique for distinguishing vessel lumen from the lumen of fiber and parenchyma ray, was first tested experimentally by assessing a threshold of the area, the circularity and the roundness. The proportion of tension wood (%) was measured based on the black and white method, by counting the black pixels in the monochrome images of the wood. Information about the accuracy and the reliability of numerical image analysis is presented in the supplementary material (**Supplementary Comment 2**). Average vessel area Ā is generally reported separately for each of these ring types (Zanne et al., 2010), however, we did not separate vessels from springwood and summer wood growth rings due to the technical difficulty of distinguishing between them among different growth rings.

#### 2.4.3 Histologic variables investigated

The variables assessed for each stem section were: the proportion (%) of xylem per stem section (xylem area was calculated by subtracting the pith area from the cross-sectional area), the proportion of tension wood (xylem black stained area divided by the xylem area), the proportion of vessels per secondary xylem, the proportion of fiber and ray cells per secondary xylem, the density of vessels per unit area (N), the density of fiber and parenchyma ray cells per unit area, the vessel to fiber density ratio (%) and the vessel to fiber area ratio.

Data on individual vessel lumen area from each stem section and for all sections were used to calculate average vessel lumen area (μm^2^), average vessel lumen diameter (D) (μm), theoretical hydraulic conductivity K_h_ within cross section area (calculated using the modified Hagen–Poiseuille law whereby conduit diameter corresponds to the vessel lumen diameter (D) (Tyree and Ewers, 1991), and theoretical specific hydraulic conductivity (K_S_) calculated by normalizing K_h_ by stem section xylem area ‘Xylem area’ (i.e. scaling data such that the hydraulic conductance of stem section with different area can be compared on the base of their water transport efficiency (Quintana-Pulido et al., 2018) (**Table 1**).

**Table 1:** Hydraulic parameters measured and calculated with acronyms, units, main definition.

Variable	Acronyms	Formula	Units	Definition	References
Vessel lumen area	A		µm^2^		
Average vessel lumen area	Ā	$\sum_{i=1}^{n}\frac{A_{i}}{N}$	µm^2^	Average area of all stem section vessels	
Vessel lumen diameter	D	$\sqrt{\frac{4A}{\pi }}$	µm	Vessel lumen diameter corresponding to circle with area A (vessel lumen area)	(Scholz et al., 2013; Quintana-Pulido et al., 2018)
Average vessel lumen diameter	D̅	$\sum_{i=1}^{n}\frac{D_{i}}{N}$	µm	Average vessel lumen diameter	
Vessel density	N	*N _per Xylem area_/Xylem area*	Number mm^-2^	Density per unit area	
Theoretical hydraulic conductivity	K_h_	$\left(\frac{\pi \rho }{128\eta }\right)\sum_{i=1}^{n}\left(D_{i}^{4}\right)$	kg m Mpa^-1^ s^-1^	Conductance per unit pressure gradient of all the vessels within the cross section area, where *ρ* is the density of water (998 kg m^-3^), *η* is the dynamic viscosity of the water (10^-9^ MPa s^-1^ at 20 °C; D is the diameter (m) of each vessel and N is the number of vessels within the cross section area	(Tyree and Ewers, 1991; Tombesi et al., 2010; Scholz et al., 2013; Kotowska et al., 2015; Quintana-Pulido et al., 2018)
Specific theoretical hydraulic conductivity	K_S_	$\frac{\left(\frac{\pi \rho }{128\eta }\right)\sum_{i=1}^{n}\left(D_{i}^{4}\right)}{Xylem\;area}$	kg m^-1^ Mpa^-1^ s^-1^	Theoretical hydraulic conductivity normalized by the stem section xylem area. It represents either a measure of stem segment porosity or measure of the ‘efficiency’ of stems in conducting water	(Bucci et al., 2006; Hacke et al., 2010; Scholz et al., 2013; Kotowska et al., 2015; Quintana-Pulido et al., 2018)

Vessel density as well as theoretical specific hydraulic conductivity K_S_ were assessed per vessel lumen diameter class of 10 μm range and corresponded to a six-class group, i.e. [10-20[µm, [20-30[µm, [30-40[µm, [40-50[µm, [50-60[µm and [60-70[µm ([a-b[µm i.e. a ≤ D< b, where b is excluded from the interval set). Also calculated were the relative frequency of the density of each diameter class (in relation to the density of all the vessels), the contribution of the K_S_ of each diameter class to the total K_S_ (i.e. to the total conductance within all the conduits of a stem section) and the accumulated K_S_ as a percentage of the total K_S_. The range class was chosen based on image analysis data which show that the smallest and the largest vessel lumen area within and among all the samples were respectively 250,015 and 2999.9 µm^2^ and correspond to a diameter D of 17.8 and 61.8 µm.

Hydraulic parameters measured and calculated are presented in **Table 1**. Additional xylem-vessel parameters calculated were the vessel lumen fraction (‘F’), the non-lumen fraction (NF), the vessel vulnerability index (VI), the vessel composition index (S) and the mean hydraulic diameter (DH) and are presented in the supplementary material (**Tables S4**, **S5**).

### 2.5 Wood composition analysis

Prior to compositional analysis, 5 g of ODW milled and sieved biomass was extracted with 95% ethanol according to the NREL protocol (Sluiter et al., 2008), using a Dionex^®^ Accelerated Solvent Extractor (ASE150) (the biomass in 33 ml cell size at 100° C under a pressure of 100 bar during 3 static cycles of 5 min for each extraction). The extracted biomass was then analyzed for structural carbohydrates and lignin in accordance with Sluiter et al. (2012). All sugars were quantified by high-performance liquid chromatography ‘HPLC’ system (Shimadzu Corporation, Kyoto, Japan) with a Bio-Rad Aminex HPX-87H column and refractive index detector. The HPLC data was corrected for the standard anhydro i.e. the contribution of water to the molecule weight of sugars between the monomer and the polysaccharide form (Serapiglia et al., 2009).

### 2.6 Statistics

Analysis of variance testing was followed by multiple comparisons of means according to Tukey’s Honestly Significant Difference (HSD) (α= 0.05) using JMP statistical software version 9.0 (SAS Institute, Cary, NC), unless otherwise stated. Pearson correlations were calculated for all pairwise combinations of xylem properties and biomass composition.

## 3 Results

### 3.1 Biomass yield

**Figure 1:**
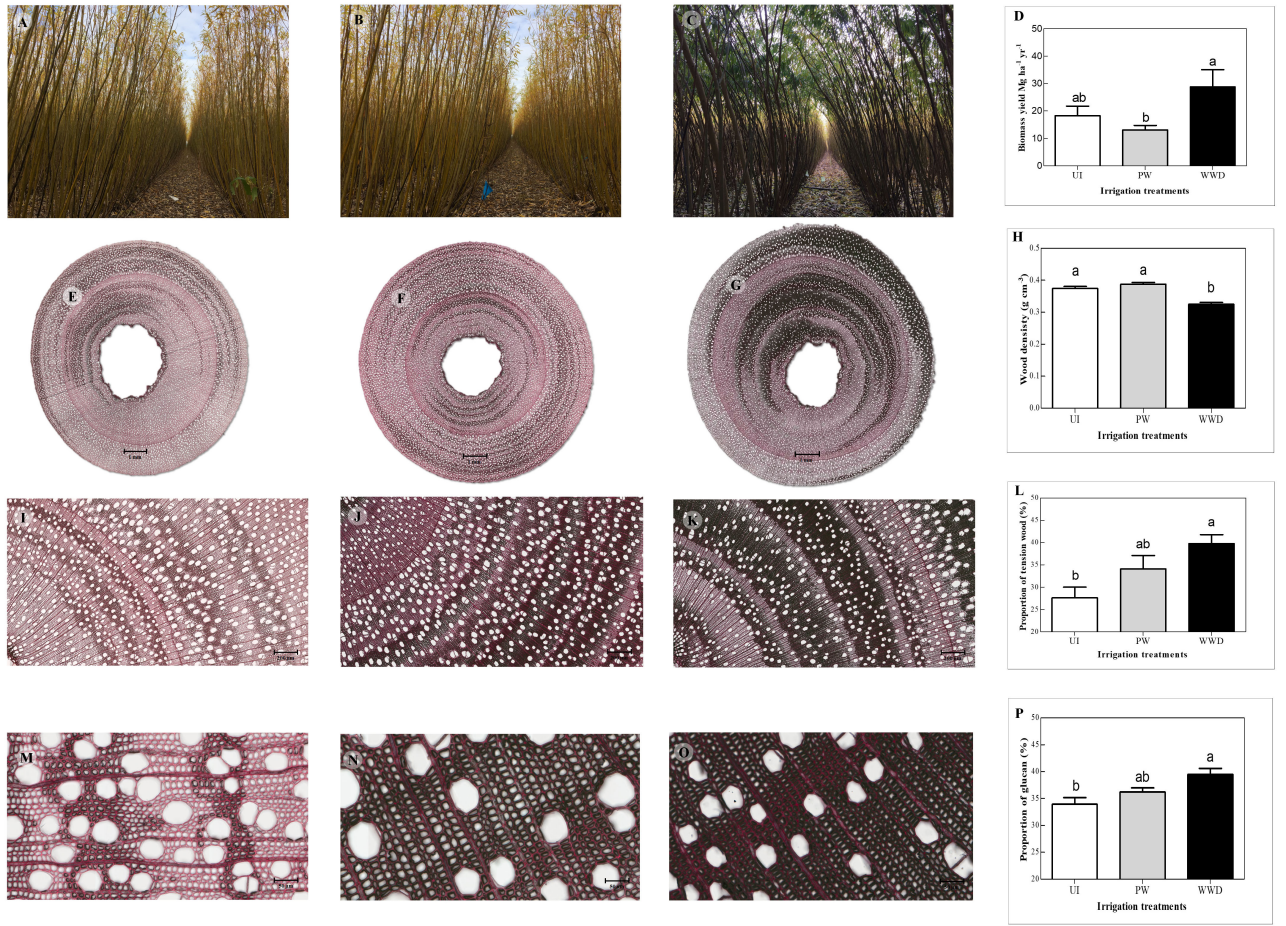
Effects of nitrogen fertilization with primary municipal wastewater on the biomass, mechanical structure and willow wood composition. **A–C**) Trees with irrigation treatments UI, PW and WWD respectively. **(E–G)** Stem section from trees with irrigation treatments UI, PW and WWD respectively (scale bar = 1mm). **(I–K)** 100x magnification of stem section region from trees with irrigation treatments UI, PW and WWD respectively (scale bar = 200 µm). **(M–O)** 500x magnification of stem section region from trees with irrigation treatments UI, PW and WWD respectively (scale bar = 50 µm). **(D)** Above ground biomass (Mg ha^-1^) of irrigation treatments UI, PW and WWD respectively (previously published results in Jerbi et al. (2020)). **(H)** Wood density (g cm^-3^) of treatments UI, PW and WWD respectively. **(L)** Proportion of tension wood (%) of irrigation treatments UI, PW and WWD respectively. **(P)** Wood proportion of glucan (%) of treatments UI, PW and WWD respectively. The results represent the average values (mean ± standard error) for each irrigation treatment. Different letters indicate significant differences according to HSD-Tukey test for the irrigation treatments (p ≤ 0.05).

After two years of growth, the total harvested biomass yields for the plants irrigated with the primary effluent WWD were higher than those of the least productive PW irrigated trees (but not statistically distinct for UI plants) with an annual yield per hectare of 18.3 ± 3.5, 13.1 ± 1.6 and 28.8 ± 6.3 Mg ha^-1^ yr^-1^ respectively for UI, PW and WWD (**Figures 1A–D**) [previously published results in Jerbi et al. (2020)].

### 3.2 Wood density and mechanical parameters

Wood density differed significantly between the different irrigation treatments, with the control UI and PW having a > 18% higher specific density than WWD with respectively 0.37, 0.39 and 0.32 g cm^-3^ (**Table 2** and **Figure 1H**).

**Table 2:** Xylem parameters and the vessels feature of *Salix miyabeana* SX67 plants under different irrigation treatments.

Treatment	Wood density (g cm^-3^)						Fibers + ray cells density (number mm^-2^)				Proportion of fibers + ray cell per secondary xylem (area %)					Proportion of tension wood per secondary xylem (area %)					Average vessel lumen area (Ā) (µm^2^)				Average vessel lumen diameter D (µm)		
UI	0.37		±		0.01 a		5695	±		90 a	81	±		0.3 b		27.6		±		2.4 b	1112	±		16 a	36.2	±	0.2 a
PW	0.39		±		0.01 a		5756	±		109 a	81	±		0.4 b		34.1		±		3.0 ab	1083	±		15 a	36.0	±	0.2 a
WWD	0.32		±		0.01 b		6243	±		70 a	84	±		0.5 a		39.8		±		2.0 a	1064	±		18 a	35.5	±	0.3 a
ANOVA *p* values	0.0126*						0.1219				0.0013*					0.0283*					0.0564				0.1143		
Treatment	Vessels density (N) (N mm^-2^)						Theoretical sapwood area-specific hydraulic conductivity K_s_ (Kg. m^-1^. Mpa ^-1^ s^-1^)				Proportion of secondary xylem per stem section (area %)					Proportion of vessels per secondary xylem (area %)					Vessels to fibers density ratio %				Vessel to fiber area ratio %		
UI	172		±		4 a		10.79	±	0.2 a		92.4	±	0.6 a		19		±		0.3 a		3.0	±	0.1 a		24	±	0.5 a
PW	173		±		3 a		9.99	±	0.3 b		94.6	±	0.4 a		19		±		0.4 a		3.0	±	0.1 a		23	±	0.6 a
WWD	152		±		4 b		8.85	±	0.4 c		95.8	±	0.6 a		16		±		0.5 b		2.4	±	0.1 b		19	±	0.8 b
ANOVA *p* values	0.0021*						<.0001*				0.0808					0.0013*					0.0033*				0.0003*		

The results represent the average values (mean ± standard error) for each irrigation treatment. For each variable, different letters indicate significant differences according to HSD-Tukey test for the irrigation treatments (p ≤ 0.05).

Although the density of fiber and ray cells did not vary significantly between UI, PW and WWD plants (**Table 2**), the proportion of fiber per secondary xylem area was higher in WWD plants compared to UI and PW plants, with respectively 84, 81 and 81% while vessel to fiber area and density ratio were significantly lower for PW plants in comparison to UI (**Table 2**).

Under all irrigation treatments, wood reacted with both dyes. A higher proportion of the wood of the wastewater irrigated plants reacted more intensely with the Chlorazol black, resulting in a larger black staining region than the other treatments, especially the UI (**Figures 1E–G**, **I–K**). Visual observations showed that for all treatments, regions of the stem section displayed the presence of an additional layer in the inner part of the wall of some fiber cells (more pronounced for WWD), contributing to their thickness (**Figures 1M–O**). Analysis of the proportion of wood presenting such formation (recognized as tension wood), showed a significant difference between irrigation treatments, especially between the UI control and the WWD plants, with the proportion respectively of 27.6, 34.1 and 39.8 % for UI, PW and WWD (**Table 2** and **Figure 1L**).

### 3.3 Stem hydraulic parameters

The average vessel lumen area did not vary significantly between treatments, measuring 1112, 1083 and 1064 µm^2^ respectively for UI, PW and WWD, nor did average vessel lumen diameter with quite similar values, i.e. 36.2, 36 and 35.5 µm (**Table 2**). Total vessel density (N mm^-2^) did differ between treatments, with WWD showing lower density than those of UI and PW with respectively 152, 172 and 173 vessels mm^-2^. As a result, the total theoretical specific hydraulic conductivity K_S_ (per stem section) did vary significantly between treatments, with UI the highest, PW intermediate and WWD significantly much lower with respectively 10.79, 9.99 and 8.85 Kg. m^-1^ Mpa^-1^ s^-1^.

**Figure 2:**
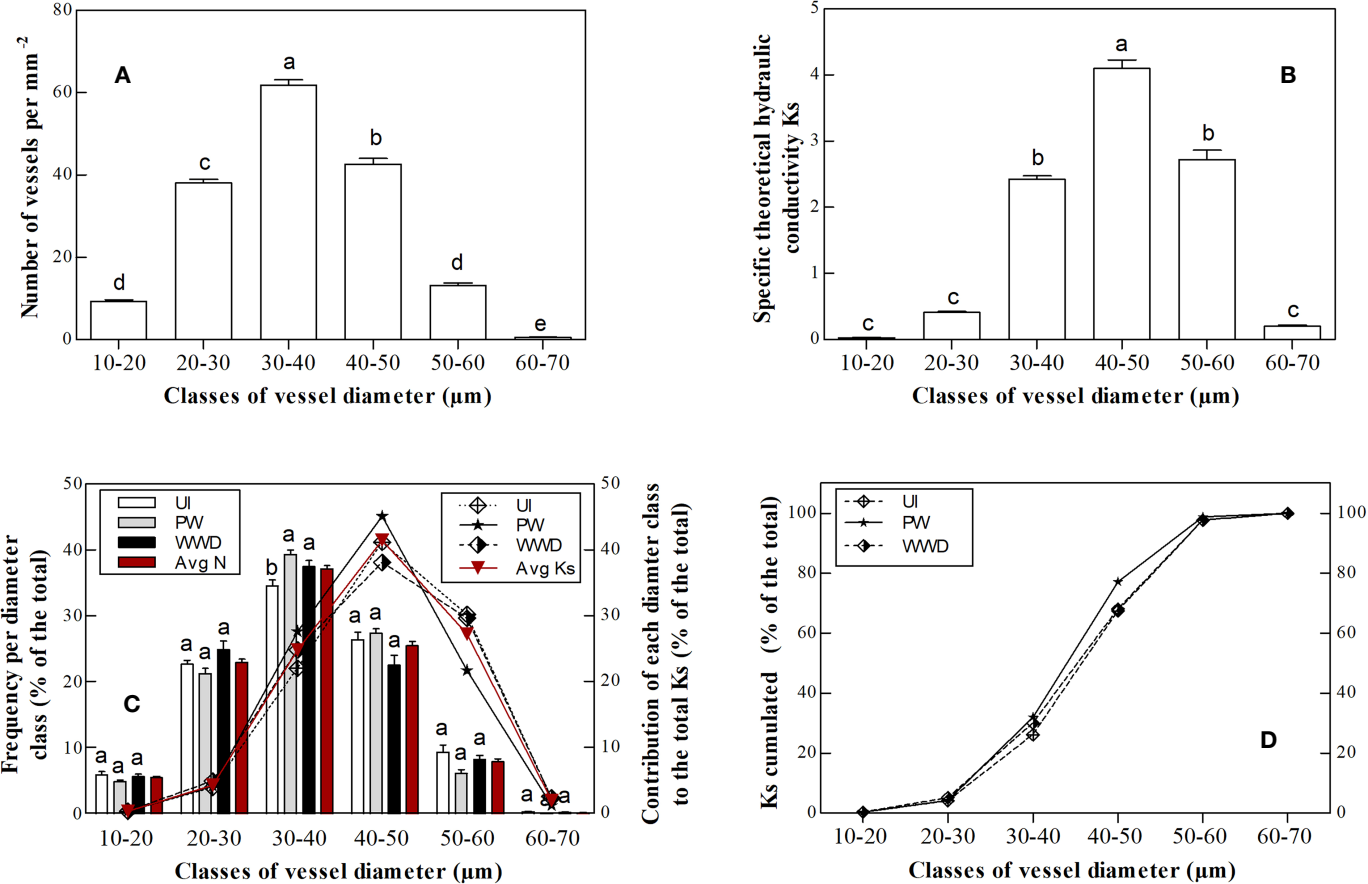
**(A)** Average vessel density (N mm^-2^) per vessel lumen diameter class, **(B)** the theoretical sapwood area-specific hydraulic conductivity KS (Kg m^-1^ pa^-1^ s^-1^) per vessel lumen diameter class, **(C)** the distributions of vessel diameters and their contribution to total hydraulic conductivity i.e. total K_S_ ( the histograms indicate vessel frequency per vessel diameter class and the lines indicate the contribution of each vessel lumen diameter class to total hydraulic conductivity K_S_), **(D)** the cumulated theoretical sapwood area-specific hydraulic conductivity KS (Kg m^-1^ pa^-1^ s^-1^) per vessel lumen diameter class . Results represent the average values (mean ± standard error) for each irrigation treatment. Different letters in the same column group indicate significant differences according to HSD-Tukey test for the irrigation treatments (p≤0.05).

For all treatments, vessel density per vessel lumen diameter range varied between the different diameter classes, i.e. 10-20, 20-30, 30-40, 40-50, 50-60 and 60-70 µm and showed a unimodal distribution, with class diameters 30-40 µm showing the highest density (**Figure 2A** and **Table S6** in the supplementary material). Similarly, K_S_ per vessel diameter range varied between the different diameter classes, with the highest K_S_ value for classes 30-40 µm, 40-50 µm and 50-60 µm (**Figure 2B** and **Table S7** in the supplementary material).

The frequency of vessel density per lumen diameter varied between the different classes, with the highest contribution to total vessel density for the group 30-40 µm, with respectively 34.7, 39.4 and 37.6% for UI, PW and WWD. Compared to PW and WWD, UI values for the 30-40 µm was significantly different. The groups 20-30 µm and 40-50 µm, which showed quite similar density frequencies, also contributed greatly to the total vessel density with 22.8, 21.4 and 25.0% respectively for UI, PW and WWD for the former group and 26.5, 27.5 and 22.7% for the latter group (**Table 3** and **Figure 2C**). For K_S_, the highest contribution was from the group 40-50 µm, with respectively 41.2, 45.1 and 38.1% for UI, PW and WWD (**Table 4**; **Figures 2C, D**).

**Table 3:** Vessel density frequency per vessel lumen diameter (D) range, i.e. the proportion of the density of each vessel class group per the density of all the vessels.

Treatment	The proportion (%) of vessels with diameter D, where a µm< D<= b µm																								
	10 ≤ D< 20				20 ≤ D<30				30 ≤ D< 40				40 ≤ D< 50				50 ≤ D< 60				60 ≤ D< 70				10 ≤ D<70 All vessels
UI	6.1	±	0.5	a	22.8	±	0.6	a	34.7	±	0.9	b	26.5	±	1.2	a	9.5	±	1.1	a	0.5	±	0.1	a	100%
PW	5.1	±	0.2	a	21.4	±	0.8	a	39.4	±	0.7	a	27.5	±	0.7	a	6.3	±	0.6	a	0.2	±	0.0	a	100%
WWD	5.8	±	0.4	a	25.0	±	1.3	a	37.6	±	0.9	a	22.7	±	1.5	a	8.4	±	0.6	a	0.4	±	0.1	a	100%
ANOVA *p* values	0.1830				0.1111				0.0051*				0.3453				0.0771				0.3462				

Results represent the average values (mean ± standard error) for each irrigation treatment. Different letters in the same diameter class group indicate significant differences according to HSD-Tukey test for the irrigation treatments (p ≤ 0.05).

**Table 4:** The ratio of the theoretical sapwood area-specific hydraulic conductivity KS (Kg m-1 pa -1 s-1) per vessel lumen diameter (D) range, i.e. the proportion of the KS of a given diameter class group to the total KS.

Treatment	The proportion (%) of the theoretical sapwood area-specific hydraulic conductivity Ks per vessel lumen diameter (D) range, where a µm< D<= b µm																								
	10 ≤ D< 20				20 ≤ D<30				30 ≤ D< 40				40 ≤ D< 50				50 ≤ D< 60				60 ≤ D< 70				10 ≤ D<70 All vessels
UI	0.3	±	0.02	a	3.9	±	0.2	a	22.0	±	1.2	b	41.2	±	2.3	a	30.2	±	2.8	a	2.3	±	0.5	a	100
PW	0.3	±	0.01	a	4.1	±	0.2	a	27.6	±	1.1	a	45.1	±	0.8	a	21.7	±	1.6	a	1.2	±	0.2	a	100
WWD	0.3	±	0.02	a	4.9	±	0.4	a	24.8	±	1.0	a	38.1	±	2.1	a	29.6	±	2.0	a	2.3	±	0.4	a	100
ANOVA *p* values	0.3397				0.0903				0.0044*				0.3717				0.1047				0.3523				

Results represent the average values (mean ± standard error) for each irrigation treatment. Different letters in the same diameter class group indicate significant differences according to HSD-Tukey test for the irrigation treatments (p ≤ 0.05).

### 3.4 Wood composition analysis

The extractives content did vary significantly between the different irrigation treatments, with the content of WWD plants, 6.8%, noticeably lower than the content of UI and PW biomass, i.e. 10.5 and 10.7% (**Table 5**).

**Table 5:** Wood composition analysis of *Salix miyabeana* SX67 plants under different irrigation treatments.

Treatment	Glucan			Xylan				Galactan				Arabinan				Mannan				Hemicellulose			
UI	33.9	±	1.2 b	11.9		±	0.5 a	1.3	±	0.06	a	1.2	±	0.1	ab	1.7	±	0.1	a	16.1	±	0.6	a
PW	36.2	±	0.7 ab	12.6		±	0.2 a	1.4	±	0.03	a	1.3	±	0.1	a	2.0	±	0.1	a	17.3	±	0.3	a
WWD	39.5	±	1.1 a	13.3		±	0.5 a	1.3	±	0.05	a	1.0	±	0.1	b	2.0	±	0.1	a	17.7	±	0.6	a
ANOVA *p* values	0.0237*			0.2900				0.2669				0.0482*				0.0789				0.2368			
Treatment	Total Sugars			ASL				AIL				Total Lignins				Extractives				Mass closure			
UI	50.0	±	1.8 b	5.2	±		0.05 a	22.5	±	0.3	a	27.7	±	0.3	a	10.5	±	0.1	a	88.2	±	1.7	a
PW	53.6	±	1.0 ab	5.1	±		0.07 a	22.1	±	0.3	a	27.1	±	0.3	a	10.7	±	0.3	a	91.4	±	1.0	a
WWD	57.2	±	1.6 a	5.3	±		0.06 a	22.8	±	0.3	a	28.1	±	0.3	a	6.8	±	0.2	b	92.1	±	1.5	a
ANOVA *p* values	0.0554			0.2268				0.2591				0.2207				0.0012*				0.2206			

Results represent the average values (mean ± standard error) for each irrigation treatment. Different letters in the same column group indicate significant differences according to HSD-Tukey test for the irrigation treatments (p ≤ 0.05).

Total lignin content as well as its acid soluble and acid insoluble fractions, i.e. ‘‘ASL’’ and ‘‘AIL’’, were statistically similar within the different irrigation treatments, with respectively 27.7, 27.1 and 28.1% for total lignin of WWD, UI and PW, 5.2, 5.1 and 5.3% for acid-soluble lignin and 22.5, 22.1 and 22.8% for acid insoluble lignin (**Table 5**).

The cellulose content of glucose differed significantly between UI, PW and WWD, with respectively 33.9, 36.2 and 39.5% (**Table 5**). Hemicellulose content did not differ between treatments UI, PW and WWD and was respectively 16.1, 17.3 and 17.7%. The content of the different hemicellulose sugar monomer components did not vary between treatments, although a statically significant difference in arabinose content between the NW and the WWD biomass was detected. Respectively, for UI, PW and WWD, the content of different monomers was 11.9, 12.6 and 13.3% for xylose, 1.3, 1.4 and 1.3% for galactose, 1.2, 1.3 and 1% for arabinose and 1.7, 2 and 2% for mannose (**Table 5**).

## 4 Discussion

We investigated the effect of treatment of a willow plantation with primary wastewater on wood properties and composition, revealing complex cell wall and stem hydraulic architecture alterations only partially similar to the well documented effects induced by high nitrogen fertilization.

### 4.1 Biomass yield

Fertigation with primary wastewater effluent containing high concentrations of nitrogen significantly increased above ground biomass by 120% on average compared to the PW-irrigated plants. The 57% biomass increase compared the control UI was not significant (**Figure 1D**) (previously published results in Jerbi et al. (2020)). Similar growth conditions may have impacted plant growth as it was observed in young *Salix nigra* plants (Pezeshki et al., 1998), another flood tolerant willow species. In previous trials using the same plantation (Guidi Nissim et al., 2015; Jerbi et al., 2015), irrigation with different loads of secondary treated wastewater effluent (compared to the primary tested here) also led to an increase in biomass production over two years of growth. The maximum yield of 18.2 Mg ha^-1^ yr^-1^ then recorded (Guidi Nissim et al., 2015; Jerbi et al., 2015) signifies 63.2% less production of biomass compared to the present study [28.8 Mg ha^-1^ yr^-1^ over a season of growth (Jerbi et al., 2020)]. No such difference between the two trials was observed on control trees than were supplement with potable water only. Such difference in the yield of fertigated plants is likely due to the higher difference in the nitrogen loads within the two trials. Thus, depending on the nature (i.e. treated or untreated municipal or industrial), wastewater load and composition (nutrient content, EC, pH) the effect of fertigation on wood biomass may lead to drastic differences in terms of yield, xylem tissue structure and wood chemical composition (Pitre et al., 2007b) and therefore broaden the opportunities for the bioproduct sector.

### 4.2 Wood density and mechanical parameters

Ranging from 0.32 to 0.39 g cm^-3^ (**Table 2** and **Figure 1H**), wood density was slightly lower in the present study than what was reported by Tharakan et al. (2003) for a group of 30 non-fertilized willow varieties (0.36 to 0.48 g cm^–3^). This was especially true for WWD plants as could be expected as a result of N fertilization (Pitre et al., 2007a; Curran et al., 2008; Hacke et al., 2010).

Although we did not investigate fiber-cells, the low wood density of WWD plants compared to UI and PW suggests a likely alteration of fiber properties (Pitre et al., 2007a; Watanabe et al., 2008; Hacke et al., 2010; Zanne et al., 2010). Indeed, wood density is related to the morphology of the cells within the secondary xylem tissue i.e. the vessels, the ray parenchyma and the fibers (Poorter et al., 2010) with the latter having the thickest cell walls and thus contributing the most. High N fertilization was reported to reduce wood density by both increasing fiber lumen and decreasing fiber cell wall thickness (Luo et al., 2005; Pitre et al., 2007a; Pitre et al., 2010; Poorter et al., 2010). Thus, besides its consequence on vessel to fiber area and density ratio, irrigation with primary wastewater may have induced such alteration of fiber cells that renders the stems more flexible to leaning under gravitation and wind circumstances (compare **Figures 1A–C**) and consequently more susceptible to the formation of tension wood. Indeed, similarly to the morphological alteration described by Pitre et al. (2007a) and Pitre et al. (2010) following N addition in poplar trees, we also observed stronger cellulose staining in the inner part of cell wall fibers of stem section of primary wastewater irrigated trees (**Figures 1M–O**), hence likely suggesting an increase of tension wood proportion within less rigid WWD treated plants.

### 4.3 Stem hydraulic parameters

Contrary to what was first hypothesized, no difference was observed between the different irrigation treatments regarding the average vessel diameter (**Table 2**) even if it should be impacted by soil water status (Hacke et al., 2010; Plavcová and Hacke, 2012) and/or increased because of nitrogen fertilization (Bucci et al., 2006; Hacke et al., 2006; Hacke et al., 2010) and thus reflected by major changes of the theoretical specific conductance K_S_ (Hacke et al., 2017).

The control UI plants did not receive water other from precipitation (about 1200 mm over the two-year trial) but showed similar hydraulic parameters (i.e. average vessel diameter and K_S_) as those of PW (**Table 2**) which received high loads of potable water i.e. 3687 mm over the same period (Jerbi et al., 2020). Because water scarcity causes xylem to exhibit narrower but more frequent vessels, this may suggest that the water was not the limiting factor for willow growth and development as both treatments UI and PW showed very similar physiological parameters as well as comparable hydraulic parameters (**Table 2**). In fact, the region where the experiment was set up is considered as a humid continental climate with generally high precipitation that may be more advantageous for a hydraulic architecture granting efficiency of water conductance (widest vessel lumen with less resistance) rather than to a ‘safe’ architecture to avoid cavitation and embolism caused by a drought that is unlikely to occur (Kotowska et al., 2015).

Intriguingly, the hydraulic conductance K_S_ was significantly lower for WWD plants than PW and UI (**Table 2**) albeit various studies reported that high N fertilization increased the production of vessels with larger lumen (Harvey and van den Driessche, 1999; Hacke et al., 2010; Hacke et al., 2017). However, for most of the diameter classes, the density of vessels was lower for WWD fertilized plants than UI and PW, especially for the groups 30-40 µm and 40-50 µm (**Figure 2A** and **Table S6** in the supplementary material). These results could be somewhat explained by the sampling strategy we used. In an effort to compare stems at equivalent developmental stages (wood that already transitioned to secondary growth before the irrigation began), samples were harvested at different heights for each treatment even though stem conducts widen basipetally and that vessel lumen diameter increases axially from the top canopy towards the roots (Larson and Isebrands, 1971; Anfodillo et al., 2013; Hacke et al., 2017).

Regardless fertigation regime, the majority of vessels were found in the diameter groups 20-30 µm, 30-40 µm and 40-50 µm which represented ~ 86% of the total vessel and contributed to ~ 68% of the hydraulic conductance K_S_ (**Tables 3**, **4**). This results is as expected for shrub species; with a higher proportion of narrow vessels than trees, i.e. ≤ 50 µm and practically no wide vessels i.e. > 200 µm (Wheeler et al., 2007; Anfodillo et al., 2013).

### 4.4 Wood composition analysis

Difference between WWD plants from one hand and UI and PW from the other shows that fertigation with N-rich wastewater decreased the extractives content of WWD biomass (**Table 5**). It is known that for a given species the composition of extractives is affected by growth conditions (Yang and Jaakkola, 2011). Fast-growing shrubs such as willows are generally associated with low bark to wood ratio. Because most of the extractives are generally located in the bark (Sassner et al., 2006; Serapiglia et al., 2009) such difference is likely to be associated with the higher growth rate of WWD plants and their lower bark content.

Several studies on willow cultivars have reported a compensatory relationship between cellulose and lignin synthesis (Guidi et al., 2009; Serapiglia et al., 2009; Ray et al., 2012; Serapiglia et al., 2013b). Although, WWD induced an increase of glucan content, lignin was similar between all treatments (**Table 5**) and rather coherent to what was reported for other willow cultivars (Serapiglia et al., 2009; Ray et al., 2012; Serapiglia et al., 2013a). Furthermore, in a study comparing several willow genotypes grown at 45 degrees to induce the formation of reaction wood, Brereton et al. (2012) also reported that glucan content did increase for plants with the most reaction wood fraction while lignin remained unchanged. Hence, the similarity between the different irrigation treatment for total lignin may suggest that irrigation with primary wastewater and/or with potable water did not affect much lignin synthesis and deposition during secondary cell wall formation (i.e. lignification phase).

**Figure 3:**
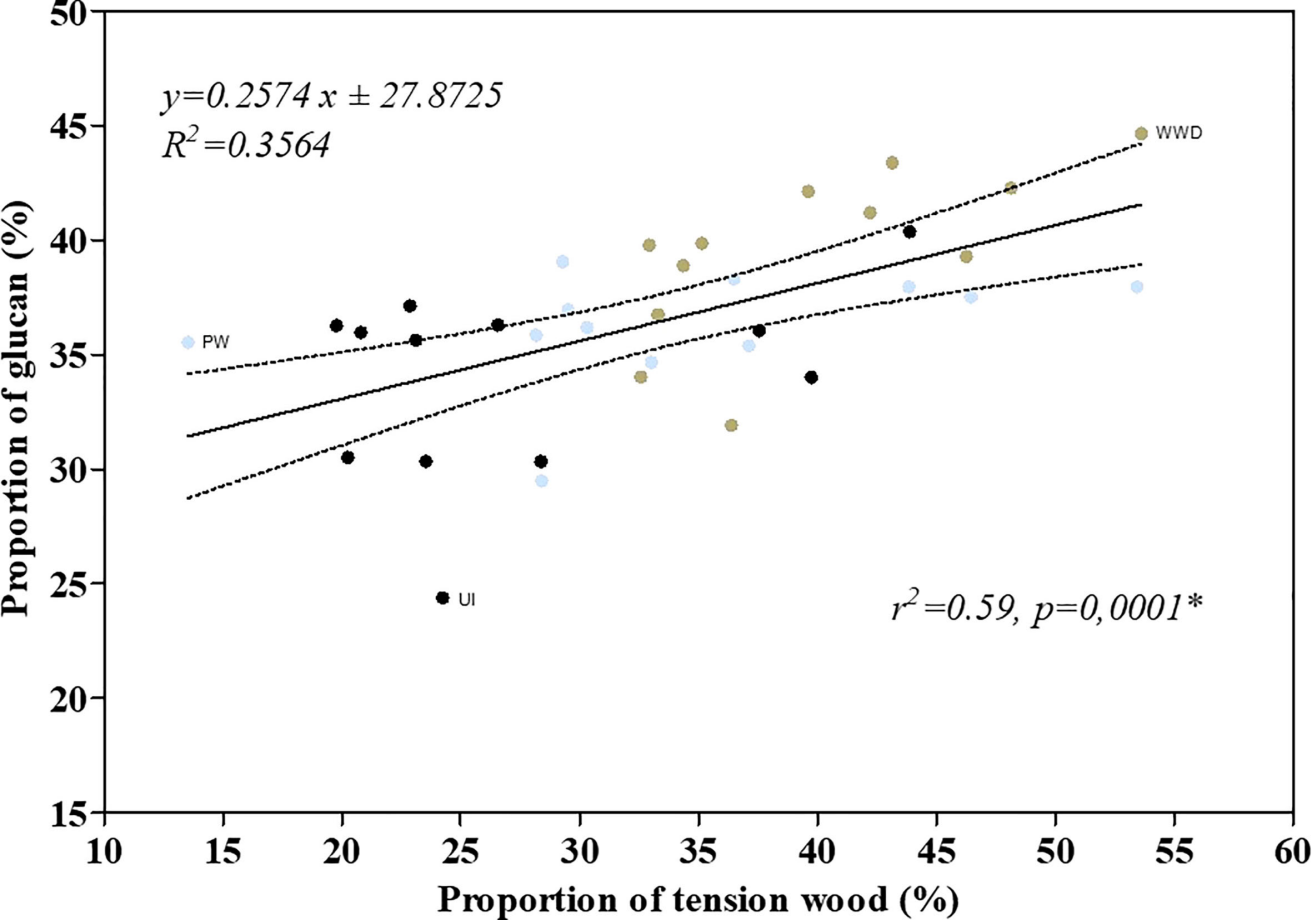
Linear regression between the tension wood proportion and the biomass glucan proportion of the cultivar *Salix miyabeana* SX67. Black, blue and brown dots refers respectively to the data of UI, PW and WWD treatments.

Alike, cellulose content (**Table 5** and **Figure 1P**) was similar to what was reported for the same cultivar as well as for other genotypes (Sassner et al., 2006; Serapiglia et al., 2008; Serapiglia et al., 2009; Ray et al., 2012; Serapiglia et al., 2013b). The glucan content did differ between UI, PW and WWD with the higher content for plants that received wastewater (**Table 5** and **Figure 1P**). High nitrogen application was previously reported to impact the development of secondary xylem of various poplar genotypes mostly by altering fiber anatomy through the deposition of an additional layer with high cellulose content in the inner part of the fiber cell lumen i.e. the G-layer (Pitre et al., 2007a; Pitre et al., 2007b; Pitre et al., 2010). Although quantitatively assaying tension wood is difficult (Brereton et al., 2011), the strong correlation (**Figure 3**, r^2^ = 0.59, p = 0.0001) between glucan content (biochemical analysis) and the proportion of tension wood (based on numerical image analysis) suggests that the increase of cellulose content is likely due to the increase of the proportion of tension wood in the case of WWD treated plants.

## 5 Conclusions

Our findings reveal that irrigation of willows with primary wastewater having high nitrogen load during two seasons of growth significantly altered wood chemical composition as well as cell wall structure. While fertigation increased the glucan content and the proportion of tension wood, it also resulted in the production of less dense wood with a significantly lower extractives fraction. This result may be of interest in the context of biofuel production and phytofiltration of municipal wastewater by SRWC. Consequently, it could provide the biofuel market with large amount of low cost-production raw material (i.e. biomass) with an increased content of glucan of higher energetic value for the conversion process (more biofuel produced per unit of biomass invested).

## Data availability statement

The raw data supporting the conclusions of this article will be made available by the authors, without undue reservation.

## Author contributions

Conceptualization, AJ, SB, FP, and ML; Methodology, AJ, KL, and P-PG; Analysis, AJ and JL; Writing – original draft preparation, AJ; Writing – review and editing, AJ, JL, FP, and ML; Project administration, FP and ML. All authors contributed to the article and approved the submitted version.
