# Supplementary material for: Irrigation with primary wastewater alters wood anatomy and composition in willow Salix miyabeana SX67
Source: Front Plant Sci. 2023 Mar 2;14:1087035. doi: 10.3389/fpls.2023.1087035 (PMC10018808; doi:10.3389/fpls.2023.1087035)
Supplement: Supplementary file 1 [file DataSheet_1.docx]

**Supplementary comment 1**

***ImageJ plug-in and analyses instructions***

**Vessels measurement**

Count the numbers of vessels and calculate the lumen area of each individual vessel within the stem section. The recognizing of vessel lumen from fiber and ray cell lumen is based on area, roundness and circularity criterion. For each high-resolution scan, the multichannel images were imported into ImageJ using the plugin Bio-Formats and the "Bio-Formats Importer" command. The multichannel images were firstly converted to single images using the “RGB Color” command and then to grayscale with the “8-bit” command. The images were then converted to pure black and white the "Auto Local Threshold". The number of vessels were assessed with the "Analyze Particles..." method using ranges of sizes and circularities respectively 250-1000 pixel and 0.1-1(unitless). The false positives were then excluded using an experimentally determined criterion of a minimal value 0.65 for the sum of a particle given roundness and circularity.

**Pith and Stem measurement**

The area of the stem section and the pith were measured using lower-resolution images. The images were treated using the same set of commands previously described to obtain pure black and white images. The pith was selected using the “Analyze Particles” with a minimum size of 30000 pixels (minimum area to be considered as pith). The whole stem was measured using the “doWand” method (ImageJ command).

**Tension wood measurement**

The amount of tension wood was measured by counting the black pixels in the monochromes images of the wood stained with the chlorazol black. Subsequent eroding and dilating of the images eliminated the areas that were less pixel-dense (corresponding to the safranine staining) while maintaining a similar superficies in the denser areas i.e. whose were stained with the chlorazol B.

**Cells lumen measurement**

The images were treated as previously described to obtain pure black and white images. The images were then smoothed using the “smooth” command and the local maxima were counted using the "Find Maxima..." command. The amount of all the cells (i.e. lumens) present within the stem section was measured by counting the local maximums in the images. The number of fibers and ray cell in the image was obtained by subtracting the number of vessels previously calculated from the number of total cells. More details about cell-counting by ‘’Maxima’’ are described in (Grishagin, 2015).

Image command

Measure Vessels density and area

- run("Bio-Formats Importer", "open=[" + input + "] color_mode=Default rois_import=[ROI manager] view=Hyperstack stack_order=XYCZT series_" + serie);
- run("RGB Color");
- run("8-bit");
- run("Auto Local Threshold", "method=Bernsen radius=25 parameter_1=0 parameter_2=0 white");
- run("Analyze Particles...", "size=" + size + " circularity=" + circ + " exclude");

**Measure the area of Pith, stem section and tension wood**

- run("Bio-Formats Importer", "open=[" + input + "] color_mode=Default rois_import=[ROI manager] view=Hyperstack stack_order=XYCZT series_" + serie);
- run("RGB Color");
- run("8-bit");

Measure the Pith area:

- run("Auto Threshold", "method=Default white")
- run("Analyze Particles...", "size=30000-Infinity display add exclude");

Measure the stem section area (including pith):

- run("Auto Threshold", "method=Default white");
- doWand(getResult("X", nResults-1),getResult("Y", nResults-1), 10, "Legacy");
- run("Measure");

Measure the tension wood area:

- run("Auto Threshold", "method=MaxEntropy white");
- run("Options...", "iterations=5 count=4 edm=8-bit do=Erode");
- run("Options...", "iterations=5 count=4 edm=8-bit do=Dilate");

**Measure cells (counting)**

- run("Bio-Formats Importer", "open=[" + input + "] color_mode=Default rois_import=[ROI manager] view=Hyperstack stack_order=XYCZT series_" + serie);
- run("RGB Color");
- run("8-bit");
- run("Smooth");
- run("Find Maxima...", "noise=10 output=Count");

**Supplementary comment 2**

***Accuracy and reliability of the analysis method***

Since vessels are not strictly cylindrical, the calculation of the specific hydraulic conductivity K_S_ based on the Hagen-Poiseuille law may overestimate the hydraulic conductivity (Tyree and Zimmermann, 2002; Quintana-Pulido *et al.*, 2018). However, the theoretical specific conductivity K_S_ calculated on the stem sections of the 2-year-old cultivar *S. miyabeana* ‘SX67’ (present study) i.e. 10.79, 9.99 and 8.85 Kg m^-1^ Mpa^-1^ s^-1^ respectively for UI, PW and WWD, was very similar to the K_S_ measured in vivo by experimental instrument XYL’EM apparatus (Bronkhorst, Montigny-les-Cormeilles, France) on similar age shoot of *Salix psammophila* i.e. 10.17±0.17 Kg  m^-1^ Mpa^-1^ s^-1^ (Li *et al.*, 2016 a; b). Thus, calculation of the theoretical hydraulic conductance based on the vessels density and size (mostly the diameter) on full stem section may be a reliable approach to estimate the hydraulic capacity of specific genotype and predict their efficiency within different environmental constraints while reducing investment in term of time and resources.

**Figure S1**: Experimental setup schematic adapted from Amiot *et al.*, (2020).


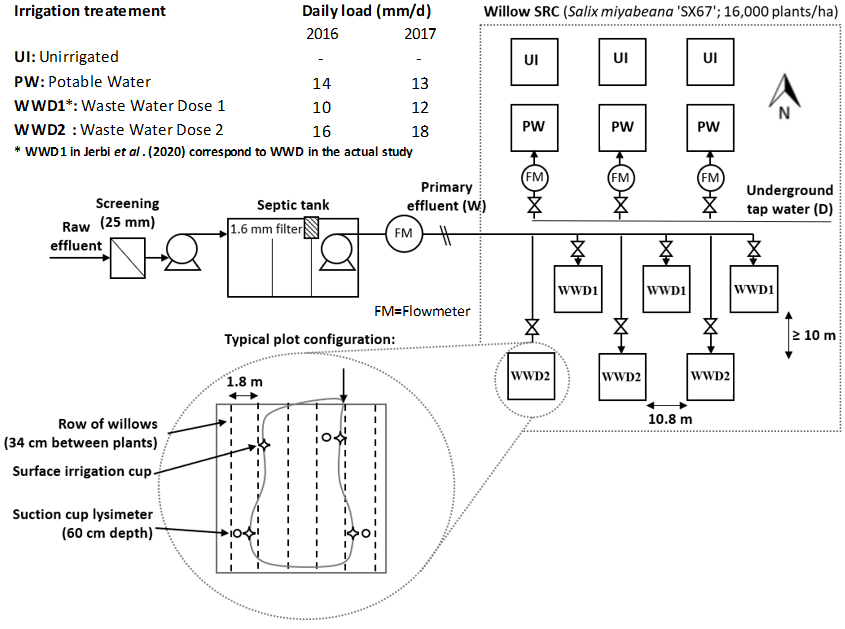


**Table S1**: Chemical characterization of the primary effluent applied during the second year of growth 2017 (From May 29^th^ to November 16^th^ 2017). The results represent the averages values (mean ± standard error) for each of the chemical components.

| Parameter |  | Symbol |  | Unit |  | Primary wastewater mean | | |
| --- | --- | --- | --- | --- | --- | --- | --- | --- |
|  |  |  |  |  |  |  | |  |
| Chemical oxygen demand |  | COD |  | mg COD L^-1^ |  | 290 | ± | 29 |
| Total nitrogen |  | TN |  | mg N L^-1^ |  | 42.1 | ± | 6.2 |
| Total ammonia |  | NH4-N |  | mg N L^-1^ |  | 20.1 | ± | 1.8 |
| Nitrates and nitrites |  | NO_X_-N |  | mg N L^-1^ |  | 0.06 | ± | 0.01 |
| Total Phosphorus |  | TP |  | mg P L^-1^ |  | 4.1 | ± | 0.6 |
| Orthophosphates |  | o-PO4 |  | mg P L^-1^ |  | 2.7 | ± | 0.7 |
| Calcium |  | Ca |  | mg Ca L^-1^ |  | 98 | ± | 6 |
| Magnesium |  | Mg |  | mg Mg L^-1^ |  | 29 | ± | 2 |
| Potassium |  | K |  | mg K L^-1^ |  | 10 | ± | 1 |
| Sodium |  | Na |  | mg Na L^-1^ |  | 145 | ± | 33 |
| Sulfate |  | SO4 |  | Mg S L^-1^ |  | 82 | ± | 9 |
| Chloride |  | Cl |  | mg Cl L^-1^ |  | 190 | ± | 57 |
| pH |  | pH |  | -- |  | 7.6 | ± | 0.1 |
| Electroconductivity |  | EC |  | dS m^-1^ |  | 1.5 | ± | 0.1 |

**Table S2**: Precipitation, water and wastewater loads during the year’s growth trial (2016 and 2017).

|  | Precipitation | | Irrigation | |
| --- | --- | --- | --- | --- |
| Year | 2016 | 2017 | 2016 | 2017 |
| Treatment | mm | mm | mm | mm |
| UI | 390 | 769 | 0 | 0 |
| PW | 390 | 769 | 1510 | 2177 |
| WWD | 390 | 769 | 1160 | 1944 |

**Table S3**: Nutrient loads through primary wastewater irrigation during the year’s growth trial (2016 and 2017).

| Parameter |  | Symbol |  |  | Nutrient loads through wastewater irrigation Kg ha^-1^ | | | | |
| --- | --- | --- | --- | --- | --- | --- | --- | --- | --- |
|  |  |  |  |  | 2016 |  | 2017 |  | 2016-2017 |
|  |  |  |  |  |  |  |  |  |  |
| Chemical oxygen demand |  | COD |  |  | 2650 |  | 5642 |  | 8292 |
| Total nitrogen |  | TN |  |  | 370 |  | 817 |  | 1187 |
| Total Kjeldahl nitrogen |  | TKN |  |  | 370 |  | 816 |  | 1186 |
| Nitrates and nitrites |  | NOx-N |  |  | 0.26 |  | 1.17 |  | 1.43 |
| Total Phosphorus |  | TP |  |  | 37 |  | 79 |  | 116 |
| Orthophosphates |  | o-PO4 |  |  | 19 |  | 52 |  | 71 |
| Calcium |  | Ca |  |  | 1647 |  | 1904 |  | 3552 |
| Magnesium |  | Mg |  |  | 476 |  | 554 |  | 1030 |
| Potassium |  | K |  |  | 151 |  | 196 |  | 347 |
| Sodium |  | Na |  |  | 4304 |  | 2827 |  | 7131 |
| Sulfate |  | SO4 |  |  | 394 |  | 1596 |  | 1990 |
| Chloride |  | Cl |  |  | 7285 |  | 3697 |  | 10982 |

**Table S4**: Supplementary vessels and hydraulic parameters measured and calculated with acronyms, units and main definition.

| **Variable** | **Acronyms** | **Formula** | **Units** | **Definition** | **References** |
| --- | --- | --- | --- | --- | --- |
| Vessel lumen fraction | F | $\bar{A}N$ | mm^2^ mm^-2^ | Fraction of the xylem occupied by the vessels lumen. Provides reliable indication of stem mechanical strength and hydraulic conductivity | (Zanne *et al.*, 2010; Scholz *et al.*, 2013) |
| Non-vessel lumen fraction | NF | $NF=1-F$ | mm^2^ mm^-2^ | Proportion of the xylem which is not vessel lumen (not accounting for other tissue lumen such as fibers and ray parenchyma cells) | (Zanne *et al.*, 2010; Scholz *et al.*, 2013) |
| Vessel composition index | S | $S=\frac{\bar{A}}{N}$ | mm^4^ | Measures the variation in the vessel composition within the transport space (vessel lumen fraction ‘F’ ) either as a change in average vessel area or in the vessels density | (Zanne *et al.*, 2010) |
| Vessel vulnerability index | VI | $VI=\frac{D}{N}$ | µm mm^-2^ | Index of plant capabilities to withstand water stress or freezing | (Scholz *et al.*, 2013) |
| Mean hydraulic diameter | D_H_ | $D_{H}=\left( \frac{\sum D^{4}}{N} \right)^{\frac{1}{4}}$ | µm | Corresponds to the lumen diameter of average Hagen-Poiseuille conductivity of given stem section i.e. the mean diameter that all vessels would have to correspond to the overall conductivity for the same numbers of vessels | (Choat *et al.*, 2011; Scholz *et al.*, 2013; Hacke *et al.*, 2017) |

**Table S5**: Data of the supplementary vessels and hydraulic parameters. Results represent the average values (mean ± standard error) for each irrigation treatment. Different letters in the same diameter class group indicate significant differences according to HSD-Tukey test for the irrigation treatments (p≤0.05).

| Treatment |  | Average vessel  perimeter (µm) | | | |  | Vessel vulnerability  index (VI) | | | |  | Vessel Lumen fraction (F) (mm^2^ mm^-2^) | | | |  | Non lumen fraction (NF) (mm^2^ mm^-2^) | | | |
| --- | --- | --- | --- | --- | --- | --- | --- | --- | --- | --- | --- | --- | --- | --- | --- | --- | --- | --- | --- | --- |
|  |  |  |  |  |  |  |  |  |  |  |  |  |  |  |  |  |  |  |  |  |
| UI |  | 155 | ± | 2.7 | a |  | 0.21 | ± | 0.006 | b |  | 0.19 | ± | 0.003 | a |  | 0.81 | ± | 0.003 | b |
| PW |  | 152 | ± | 4.4 | a |  | 0.21 | ± | 0.004 | b |  | 0.19 | ± | 0.004 | a |  | 0.81 | ± | 0.004 | b |
| WWD |  | 153 | ± | 2.0 | a |  | 0.24 | ± | 0.006 | a |  | 0.16 | ± | 0.005 | b |  | 0.84 | ± | 0.005 | a |
|  |  |  | | | |  |  | | | |  |  | | | |  |  | | | |
| ANOVA *p* values |  | 0.8726 | | | |  | 0.0068* | | | |  | 0.0013* | | | |  | 0.0013* | | | |
| Treatment |  | Vessel size to number ratio (S) (mm^4^) | | | |  | Mean hydraulic | | | |  | ∑D4 (m^4^ mm^-2^) | | | |  | Hydraulic conductivity K_h_ (Kg m Mpa ^-1^ s^-1^) | | | |
|  |  |  |  |  |  |  |  |  |  |  |  |  |  |  |  |  |  |  |  |  |
| UI |  | 6.5E-06 | ± | 2.3E-07 | ab |  | 40.0 | ± | 0.33 | a |  | 4.4E-10 | ± | 9.4E-12 | a |  | 6.2E-04 | ± | 2.3E-05 | a |
| PW |  | 6.3E-06 | ± | 1.5E-07 | b |  | 39.1 | ± | 0.26 | b |  | 4.1E-10 | ± | 1.3E-11 | b |  | 5.7E-04 | ± | 2.4E-05 | a |
| WWD |  | 7.1E-06 | ± | 2.0E-07 | a |  | 39.2 | ± | 0.30 | b |  | 3.6E-10 | ± | 1.5E-11 | c |  | 5.3E-04 | ± | 2.3E-05 | a |
|  |  |  | | | |  |  | | | |  |  | | | |  |  | | | |
| ANOVA *p* values |  | 0.0252* | | | |  | 0.0152* | | | |  | <.0001* | | | |  | 0.1206 | | | |

**Table S6**: Vessel density (N mm-2) per vessel lumen diameter (D) class. Results represent the average values (mean ± standard error) for each irrigation treatment. Different letters in the same diameter class group indicate significant differences according to HSD-Tukey test for the irrigation treatments (p≤0.05).

|  |  | Density of vessels with diameter D, where a µm < D <= b µm | | | | | | | | | | | | | | | | | | | | | | | | | | | | | | | | | |
| --- | --- | --- | --- | --- | --- | --- | --- | --- | --- | --- | --- | --- | --- | --- | --- | --- | --- | --- | --- | --- | --- | --- | --- | --- | --- | --- | --- | --- | --- | --- | --- | --- | --- | --- | --- |
| Treatment |  | 10 ≤ D < 20 | | | |  | 20 ≤ D <30 | | | |  | 30 ≤ D < 40 | | | |  | 40 ≤ D < 50 | | | |  | 50 ≤ D < 60 | | | |  | 60 ≤ D < 70 | | | |  | 10 ≤ D <70  All vessels | | | |
|  |  |  |  |  |  |  |  |  |  |  |  |  |  |  |  |  |  |  |  |  |  |  |  |  |  |  |  |  |  |  |  |  |  |  |  |
| UI |  | 10 | ± | 0.7 | a |  | 39 | ± | 1.7 | a |  | 60 | ± | 2.5 | b |  | 46 | ± | 2.5 | a |  | 16 | ± | 1.5 | a |  | 0.8 | ± | 0.2 | a |  | 172 | ± | 4 | a |
| PW |  | 9 | ± | 0.4 | a |  | 37 | ± | 1.5 | a |  | 68 | ± | 1.7 | a |  | 48 | ± | 1.4 | a |  | 11 | ± | 1.0 | a |  | 0.4 | ± | 0.1 | a |  | 173 | ± | 3 | a |
| WWD |  | 9 | ± | 0.6 | a |  | 38 | ± | 1.9 | a |  | 57 | ± | 2.3 | b |  | 35 | ± | 2.6 | a |  | 13 | ± | 1.1 | a |  | 0.6 | ± | 0.1 | a |  | 152 | ± | 4 | b |
|  |  |  |  |  |  |  |  |  |  |  |  |  |  |  |  |  |  |  |  |  |  |  |  |  |  |  |  |  |  |  |  |  |  |  |  |
| ANOVA *p* values |  | 0.1698 | | | |  | 0.6008 | | | |  | 0.0113* | | | |  | 0.0974 | | | |  | 0.0807 | | | |  | 0.3769 | | | |  | 0.0023* | | | |

**Table S7**: Theoretical sapwood area-specific hydraulic conductivity KS (Kg. m-1.pa-1 s-1) per vessel lumen diameter (D) class. Results represent the average values (mean ± standard error) for each irrigation treatment. Different letters in the same diameter class group indicate significant differences according to HSD-Tukey test for the irrigation treatments (p≤0.05).

|  |  | The theoretical sapwood area-specific hydraulic conductivity Ks per vessel  lumen diameter (D) range, where a µm < D <= b µm | | | | | | | | | | | | | | | | | | | | | | | | | | | | | | | | | |
| --- | --- | --- | --- | --- | --- | --- | --- | --- | --- | --- | --- | --- | --- | --- | --- | --- | --- | --- | --- | --- | --- | --- | --- | --- | --- | --- | --- | --- | --- | --- | --- | --- | --- | --- | --- |
| Treatment |  | 10 ≤ D < 20 | | | |  | 20 ≤ D <30 | | | |  | 30 ≤ D < 40 | | | |  | 40 ≤ D < 50 | | | |  | 50 ≤ D < 60 | | | |  | 60 ≤ D < 70 | | | |  | 10 ≤ D <70  All vessels | | | |
|  |  |  |  |  |  |  |  |  |  |  |  |  |  |  |  |  |  |  |  |  |  |  |  |  |  |  |  |  |  |  |  |  |  |  |  |
| UI |  | 0.03 | ± | 0.002 | a |  | 0.42 | ± | 0.02 | a |  | 2.4 | ± | 0.1 | b |  | 4.4 | ± | 0.2 | a |  | 3.3 | ± | 0.3 | a |  | 0.3 | ± | 0.05 | a |  | 10.8 | ± | 0.2 | a |
| PW |  | 0.03 | ± | 0.001 | a |  | 0.40 | ± | 0.02 | a |  | 2.7 | ± | 0.1 | a |  | 4.5 | ± | 0.1 | a |  | 2.2 | ± | 0.2 | a |  | 0.1 | ± | 0.02 | a |  | 10.0 | ± | 0.3 | b |
| WWD |  | 0.03 | ± | 0.002 | a |  | 0.42 | ± | 0.02 | a |  | 2.2 | ± | 0.1 | b |  | 3.4 | ± | 0.2 | a |  | 2.6 | ± | 0.2 | a |  | 0.2 | ± | 0.04 | a |  | 8.8 | ± | 0.4 | c |
|  |  |  |  |  |  |  |  |  |  |  |  |  |  |  |  |  |  |  |  |  |  |  |  |  |  |  |  |  |  |  |  |  |  |  |  |
| ANOVA *p* values |  | 0.1761 | | | |  | 0.6715 | | | |  | 0.0034* | | | |  | 0.1155 | | | |  | 0.1064 | | | |  | 0.3787 | | | |  | <.0001* | | | |
